# A multicenter, phase I, pharmacokinetic study of osimertinib in cancer patients with normal renal function or severe renal impairment

**DOI:** 10.1002/prp2.613

**Published:** 2020-06-22

**Authors:** Karthick Vishwanathan, Inmaculada Sanchez‐Simon, Bhumsuk Keam, Nicolas Penel, Maria de Miguel‐Luken, Doris Weilert, Andrew Mills, Marcelo Marotti, Martin Johnson, Alain Ravaud

**Affiliations:** ^1^ Clinical Pharmacology and Safety Science R&D, AstraZeneca Boston MA USA; ^2^ Departamento de Oncología Hospital Universitario Virgen del Rocío Seville Spain; ^3^ Department of Internal Medicine Seoul National University Hospital Seoul South Korea; ^4^ Department of Medicine Centre Oscar Lambret and Lille University (Univ. Lille) Lille France; ^5^ START Madrid‐Centro Integral Oncológico Clara Campal Sanchinarro University Hospital Madrid Spain; ^6^ IQVIA Overland Park KS USA; ^7^ Biometrics and Information Sciences AstraZeneca Cambridge UK; ^8^ Clinical Pharmacology and Safety Science R&D, AstraZeneca Cambridge UK; ^9^ Medical Oncology Department Bordeaux University Hospital Saint‐André Hospital Bordeaux France

**Keywords:** epidermal growth factor receptors, kidney, non‐small cell lung cancer, osimertinib, pharmacokinetics, renal disposition, tyrosine kinase inhibitors

## Abstract

Osimertinib is a third‐generation, irreversible, oral epidermal growth factor receptor (EGFR)‐tyrosine kinase inhibitor (TKI) that potently and selectively inhibits both EGFR‐TKI sensitizing and EGFR T790M and has demonstrated efficacy in non‐small cell lung cancer (NSCLC) central nervous system metastases. In this phase I study, we assessed the effects of normal renal function (NRF) and severe renal impairment (SRI) on the pharmacokinetics (PK) of osimertinib in patients with solid tumors. Part A: patients with NRF (creatinine clearance [CrCL] ≥90 mL/min), and SRI, (CrCL <30 mL/min), received a single 80‐mg oral dose of osimertinib and standard PK measures were assessed. Part B: patients with SRI were treated for 3 months to obtain safety data, if deemed clinically appropriate. The geometric mean osimertinib plasma concentrations were higher in patients with SRI (n = 7) vs NRF (n = 8) and were highly variable. Osimertinib exposure based on *C*_max_ and area under the plasma concentration‐time curve, was 1.19‐fold (90% CI: 0.6, 2.0) and 1.85‐fold (90% CI: 0.9, 3.6), respectively, higher for patients with SRI vs patients with NRF, with no clear correlation between CrCL and exposure. No new safety signals were identified after 12 weeks of osimertinib 80 mg continuous dosing. PK parameters pooled across this study and other phase I, II, and III osimertinib clinical studies (exploratory population PK analysis), showed minimal correlation between CrCL and total clearance. In conclusion, no dose adjustment is required for osimertinib for patients with SRI.

**Abbreviations**
%GCV: percent geometric coefficient of variation
AE: adverse event
AESI: AEs of special interest
ALT: alanine aminotransferase
ANOVA: analysis of variance
AST: aspartate aminotransferase
AUC_(0‐_*_t_*_)_: area under the plasma concentration‐time curve from zero to the last quantifiable time point
AUC: area under the plasmaconcentration‐time curve
AUC_ss_: area under the plasma concentration‐time curve at steady state
AZ5104: N‐[2‐[2‐(dimethylamino)ethyl‐methylamino]‐5‐[[4‐(1Hindol‐3‐yl)pyrimidin‐2‐yl]amino]‐4‐methoxyphenyl]prop‐2‐enamide
AZ7550: N‐[4‐methoxy‐5‐[[4‐(1‐methylindol‐3‐yl)pyrimidin‐2‐yl]amino]‐2‐[methyl‐[2‐(methylamino)ethyl]amino]phenyl]prop‐2‐enamide
C‐G: Cockcroft‐Gault
CI: confidence interval
CL/F: apparent plasma clearance
CL_R_: renal clearance
*C*_max_: maximum plasma concentration
CrCL: creatinine clearance
EGFR: epidermal growth factor receptor
EGFRm: EGFR‐TKI sensitizing mutations
EMA: European Medicines Agency
FDA: Food and Drug Administration
LS: least square
MR: metabolite to parent ratio
NRF: normal renal function
NSCLC: non‐small cell lung cancer
PK: pharmacokinetics
SAE: serious adverse event
SD: standard deviation
SRI: severe renal impairment
*t*_1/2lz_: terminal half‐life
TKI: tyrosine kinase inhibitor
*t*_max_: time of maximum concentration
*V_z_* /*F*: apparent volume of distribution

## 1 INTRODUCTION

Osimertinib is a third‐generation, irreversible, oral epidermal growth factor receptor (EGFR)‐tyrosine kinase inhibitor (TKI) that potently and selectively inhibits both EGFR‐TKI sensitizing (EGFRm) and EGFR T790M and has demonstrated efficacy in non‐small cell lung cancer (NSCLC) CNS metastases.^1^, ^2^, ^3^, ^4^, ^5^, ^6^

Osimertinib is currently approved in 84 countries, for the treatment of patients with locally advanced or metastatic EGFR T790M mutation‐positive NSCLC, and in 75 countries for use as first‐line treatment of patients with locally advanced or metastatic NSCLC whose tumors have EGFR exon 19 deletion or p.Leu858Arg EGFR mutations (with country‐specific variations).^7^, ^8^, ^9^

In patients with EGFRm NSCLC, osimertinib exposure, maximum plasma concentration (*C*_max_), and area under the concentration‐time curve (AUC) increase with dose proportionally from 20 to 240 mg/day after single and multiple dosing.^10^, ^11^ The mean half‐life of osimertinib is ~48 hours and visual observations of trough levels indicate steady‐state is generally achieved by 15 days of dosing, consistent with single‐dose pharmacokinetics (PK).^10^

In vitro reaction phenotyping studies indicate that CYP3A4/5 are the principal cytochrome enzymes responsible for the metabolism of osimertinib and its two most abundant metabolites, AZ5104 (N‐[2‐[2‐(dimethylamino)ethyl‐methylamino]‐5‐[[4‐(1H‐indol‐3‐yl)pyrimidin‐2‐yl]amino]‐4‐methoxyphenyl]prop‐2‐enamide) and AZ7550 (N‐[4‐methoxy‐5‐[[4‐(1‐methylindol‐3‐yl)pyrimidin‐2‐yl]amino]‐2‐[methyl‐[2‐(methylamino)ethyl]amino]phenyl]prop‐2‐enamide), with a smaller contribution by renal clearance.^12^ Both metabolites are potentially active; however, they each circulate at levels ~10% of that seen with osimertinib.^10^ In a ^14^C‐osimertinib mass balance study, after a single 20 mg oral dose, ~68% of the dose was eliminated in feces and 14% in urine, with unchanged osimertinib accounting for <2% (0.8% in urine and 1.2% in feces) of the dose.^8^, ^9^, ^12^ In a hepatic impairment study, osimertinib exposure was not increased due to mild or moderate hepatic impairment (Child Pugh A or B).^13^

Although osimertinib urinary excretion is low and renal impairment is not expected to have a significant impact on the PK of osimertinib, it has been observed that severe renal impairment (SRI) can impact the exposure of many compounds that are not primarily eliminated renally.^14^

In a previous population PK analysis of osimertinib in 593 patients with mild renal impairment (creatinine clearance [CrCL] 60 to <90 mL/min), 254 patients with moderate renal impairment (CrCL 30 to <60 mL/min), five patients with severe renal impairment (CrCL 15 to <30 mL/min) and 502 patients with normal renal function (NRF; CrCL ≥90 mL/min), osimertinib exposures were similar.^8^ Across osimertinib clinical trials, which were part of this population PK analysis, data from patients with SRI (n = 5) were very limited and at the time of the initiation of this clinical study, it was even lower (n = 3). As osimertinib may be used by patients who suffer from varying degrees of renal impairment, it is important to define the effects on the PK of osimertinib, to determine whether it is necessary to develop dose adjustment recommendations and, thereby, ensure appropriate use.

Here, we report the results of Part A (single‐dose PK phase) and Part B (continued dosing 12‐week safety phase in patients with SRI) of a three‐part phase I trial (NCT02923947) designed to characterize the impact of SRI on the PK of osimertinib and its metabolites (AZ5104 and AZ7550) in patients with advanced solid tumors.

## 2 MATERIALS AND METHODS

### 2.1 Trial design

**FIGURE 1:**
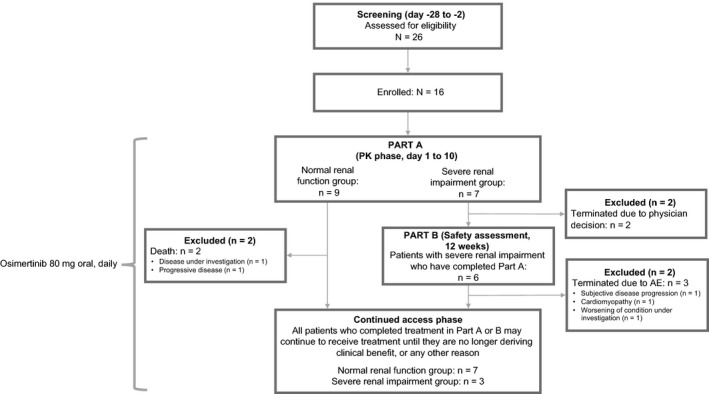
Study design and patient disposition

This was a phase I, open‐label, non‐randomized, multicenter, three‐part study (Part A, Part B, and continued access) in patients with advanced solid tumors. All patients were included in Part A; following this, those with SRI were eligible for Part B. Patients with NRF who completed Part A, and patients with SRI who completed Part B, could be included in the continued access phase if the investigator and/or the patient thought they were receiving clinical benefit Figure 1. Herein, we report the results of Part A and Part B.

The primary objective of the study was to characterize the effect of SRI on the PK of a single oral dose of osimertinib, 80 mg (Part A) in patients with advanced solid tumors. Secondary objectives included characterization of the effect of SRI on the PK of osimertinib metabolites (AZ5104 and AZ7550) after a single dose of osimertinib and to investigate the safety and tolerability of single dose and continuous dosing of osimertinib in the same patient groups (Part A and Part B). SRI was defined as having a CrCL of <30 mL/min, as measured by the Cockcroft‐Gault (C‐G) formula [see Supplementary Section and Table S1]).^15^ NRF was defined as CrCL >90 mL/min.

The study was performed in accordance with the ethical principles that have their origin in the Declaration of Helsinki and that are consistent with International Council for Harmonisation (ICH)/Good Clinical Practice (GCP), applicable regulatory requirements and the AstraZeneca policy on Bioethics. Protocols were reviewed and approved by an independent ethics committee or institutional review boards in each participating country before implementation. Data underlying the findings described in this article may be obtained in accordance with AstraZeneca's data sharing policy described at https://astrazenecagrouptrials.pharmacm.com/ST/Submission/Disclosure.

### 2.2 Participants

Patients were aged ≥18 years, had Eastern Cooperative Oncology Group performance status ≤2, and histological or cytological confirmation of a solid, malignant tumor (excluding lymphoma) that was refractory to standard therapies or for which no standard therapies exist. Patients with asymptomatic, stable central nervous system metastases not requiring steroids for at least 4 weeks prior to the start of study treatment were permitted. Previous cancer treatments had to be completed before study entry (see Supplementary Section). Patients on dialysis and patients who had undergone prior kidney transplantation were ineligible to participate.

The inclusion criterion for NRF was as previously defined, at screening. For renal impairment groups, patients had to have stable SRI, as previously defined, for at least 2 months before the start of the study. For patients with SRI, the use of concurrent medication known to affect CrCL (for example, cephalosporin antibiotics, ascorbic acid, trimethoprim, cimetidine, quinine, nephrotoxic drugs) within 7 days of the first dose of study treatment was prohibited.

The demographics (age, body mass index, sex) of patients with NRF were matched as closely as possible to patients with SRI. Hepatic function was evaluated at baseline and throughout the study, by analysis of alanine aminotransferase (ALT), aspartate aminotransferase (AST), and bilirubin levels.

### 2.3 Safety and tolerability

Safety assessments included adverse event (AE) reporting graded by Common Terminology Criteria for Adverse Events (version 4.0), physical examination, vital signs, electrocardiogram (ECG), ophthalmological examination, clinical chemistry, hematology, and urinalysis.

### 2.4 Sample collection and bioanalysis

Plasma samples were collected for PK analysis predose and at 1, 2, 4, 6, 8, 10, 24, 48, 72, 120, 168, and 216 hours post dose, and for protein‐binding analysis at 6, 24, 48, and 168 hours. Pooled urine was collected over the interval of 0‐24 hours. Sample bioanalysis was performed by Covance Laboratories (Harrogate, UK) using validated bioanalytical methods.

Further sample collection and bioanalysis methods can be found in the Supplementary Section.

### 2.5 Pharmacokinetic analysis

The PK parameters were derived using non‐compartmental methods with Phoenix^®^ WinNonlin^®^ version 8.0 (Pharsight Corp., A Certara Company) and/or SAS^®^ version 9.4 (SAS Institute, Inc). The actual elapsed time from dose administration was used in the final plasma PK parameter calculations. All descriptive and inferential statistical computations were performed using SAS^®^ version 9.4. Calculation of PK parameters and statistical analysis of PK and safety data was performed by IQVIA™ (formerly QuintilesIMS) under the direction of the Biostatistics Group, AstraZeneca, using SAS^®^ version 9.4.

### 2.6 Statistical methods

To provide adequate PK information and to assess the effects of SRI on the PK of osimertinib, while exposing as few patients as possible to the investigational treatment and procedures, an appropriate number of patients needed to be enrolled. Guidance from the European Medicines Agency (EMA), Committee for Medicinal Products for Human use suggests that a population of six to eight patients per group is required to provide adequate PK data,^16^ thus, according to this recommendation, we planned to enrol a minimum of six assessable patients per renal function group. To allow for completion of at least six evaluable patients per renal function group (severe impairment and normal), a total of eight patients per renal function group were recruited.

The PK analysis set was defined as all patients who received an osimertinib dose and had at least one post dose quantifiable plasma osimertinib or metabolite (AZ5104 or AZ7550) concentration without important protocol deviations/violations that could affect PK evaluation. The safety analysis set included all patients who received at least one dose of osimertinib.

Further statistical methods can be found in the Supplementary Section.

### 2.7 Population pharmacokinetic analysis

An additional assessment of the impact of renal impairment on the PK of osimertinib was performed using a population PK analysis and the surrogate marker of CrCL as determined by the C‐G formula. Previously published population PK analysis methods were updated with additional clinical data from this study.^17^ The population PK data set included patient data from phase I, II, and III osimertinib trials AURA (NCT01802632),^11^, ^18^ AURA2 (NCT02094261),^19^ AURA3 (NCT02151981),^2^ and FLAURA (NCT02296125).^3^ Details of the studies included in the population PK dataset are provided in Supplementary Section and Table S2. A linear regression model was conducted, to assess the relationship between empirical Bayes estimate for total apparent plasma clearance (CL/F) and baseline CrCL including patients with SRI’s data from this study (n = 7 SRI patients) to the population PK dataset (n = 1364, of which there were five patients with SRI). Statistical comparisons of osimertinib exposure parameters (area under the plasma concentration‐time curve at steady state [AUC_ss_]) from the population PK analysis set (n = 1364) combined with this study (n = 16) were also performed and the results were summarized. Furthermore, to account for the small sample size of patients with SRI, matched comparison analysis was performed. In this analysis, 100 random clinical trial datasets of 12 normal renal status subjects were sampled, matching for age, body mass index, and sex covariates from the pool of population PK and this study data, and compared with 12 severe renally impaired subjects (seven from this study, and five as mentioned previously). For each clinical trial dataset, analysis of variance (ANOVA) statistical analysis was performed and mean differences between groups and respective confidence intervals for each dataset were calculated.

## 3 RESULTS

### 3.1 Patients

In this analysis, 26 patients were screened, and 16 were enrolled and assigned to treatment Figure 1. Three patients (all in the normal group) had important protocol deviations during the study, all were included in the safety analyses, two were included in the PK analyses. Additional information on important protocol deviations can be found in the Supplementary Section. The PK analysis set included 15 patients; the safety analysis set for Part A included 16 patients; Part B (SRI) included six patients Figure 1.

Baseline demographics and disease characteristics are summarized in Table 1. The median (range) age of patients in the SRI group was higher (71 [68‐88] years) than the normal group (63 [59‐73] years); this difference was within the protocol‐defined tolerance for matched patients. Two more male patients were included in the normal group (five patients [56%]) vs the SRI group (three patients [43%]). Mean body mass index was similar between the two groups: 26.5 kg/m^2^ and 25.5 kg/m^2^ in the normal and severe groups, respectively. The most common primary tumor location was the lung (four of nine patients in the normal group and three of seven in the severe renal function group). Mutation status of patients or efficacy was not evaluated in this study. The baseline liver function indicated that there were no cases of hepatic disorders during the study. Hepatic function was not significantly impacted in patients with SRI vs normal.

**TABLE 1:** Baseline demographics and disease characteristics (Safety analysis set)

	Part A			Part B
	Normal renal function^e^ (N = 9)	Severe renal impairment^f^ (N = 7)	Total (N = 16)	Severe renal impairment^f^ (N = 6)
Age (y), median (range)	63 (59, 73)	71 (68, 88)	68 (59, 88)	72 (68, 88)
Sex, n (%)				
Male	5 (56)	3 (43)	8 (50)	3 (50)
Female	4 (44)	4 (57)	8 (50)	3 (50)
Race^a^, n (%)				
White	4 (67)	5 (71)	9 (69)	5 (83)
Asian	2 (33)	2 (29)	4 (31)	1 (17)
ECOG PS, n (%)				
0 (normal activity)	5 (56)	2 (29)	7 (44)	2 (33)
1 (restricted activity)	4 (44)	3 (43)	7 (44)	2 (33)
2 (in bed >50% of the time)	0	2 (29)	2 (13)	2 (33)
Primary tumor location, n (%)				
Lung	4 (44)	3 (43)	7 (44)	3 (50)
Kidney	0	3 (43)	3 (19)	2 (33)
Biliary tract	1 (11)	0	1 (6)	0
Breast	1 (11)	0	1 (6)	0
Colon	1 (11)	0	1 (6)	0
Pancreas	1 (11)	0	1 (6)	0
Supraglottis	1 (11)	0	1 (6)	0
Skin	0	1 (14)	1 (6)	1 (17)
Overall disease classification				
Metastatic^b^	9 (100)	7 (100)	16 (100)	6 (100)
Locally advanced^c^	0	0	0	0
Both^d^	7 (78)	4 (57)	11 (69)	4 (67)

Abbreviations

ECOG, Eastern Cooperative Oncology Group; PS, performance status; SD, standard deviation.

^a^ Data are “not applicable” for the 3 patients from France, due to local laws.

^b^ Patient has any metastatic site of disease.

^c^ Patient has only locally advanced sites of disease.

^d^ Patient has both locally advanced and metastatic sites of disease.

^e^ Normal renal function creatinine clearance (CrCL) ≥90 mL/min.

^f^ Severe renal impairment CrCL <30 mL/min.

### 3.2 Osimertinib pharmacokinetics

**FIGURE 2:**
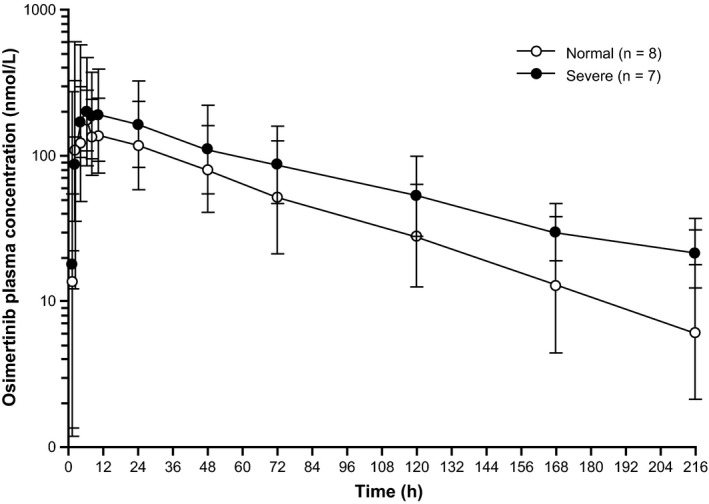
Geometric mean plasma concentration of osimertinib by renal function group (semi‐logarithmic scale; pharmacokinetics analysis set). Geometric mean standard deviation expressed in the error bars as the exponential of (mean of the log concentration ± the standard deviation of the log concentration). Normal renal function creatinine clearance (CrCL) ≥90 mL/min; Severe renal impairment CrCL of <30 mL/min. One patient with a CrCL of 80 mL/min at screening was excluded from this summary

For patients with SRI, geometric mean osimertinib concentrations were higher when compared with patients with NRF; however, there was a large overlap across both cohorts at each time point in the osimertinib concentrations Figure 2. Individual and geometric mean AUC and *C*_max_ for each renal function group are shown in Supplementary Section and Figure S1 Part A and B, respectively. On average, PK parameters showed a higher AUC and *C*_max_ for osimertinib for patients with SRI vs those with NRF Table 2. Osimertinib exposure, based on *C*_max_ and AUC, was 1.19‐fold and 1.85‐fold, respectively, for patients with SRI relative to patients with NRF. Because of the limited sample size, the 90% CIs were wide and included unity (1.00 Table 2).

**TABLE 2:** Pharmacokinetic parameters statistical comparisons of osimertinib, AZ5104, and AZ7550 for each renal function group^a^ (Pharmacokinetic analysis set)

Pharmacokinetic parameter	Normal renal function	Severe renal impairment
Osimertinib	(N = 8)	(N = 7)
AUC (nmol/L·h), geometric LS mean (%GCV)	11 070 (87.5)	20 460 (69.4)^b^
Comparison, ratio, % (90% CI)	184.8 (93.9, 363.8)	
*C*_max_ (nmol/L), geometric LS mean (%GCV)	195.5 (57.7)	233.4 (74.8)
Comparison, ratio, % (90% CI)	119.4 (68.9, 206.9)	
*t*_max_ (h), median (min, max)	5.0 (2.0, 6.1)	6.0 (4.0, 10.1)
*t*_1/2λz_ (h), mean (SD)	49.3 (13.0)	71.4 (14.8)^b^
CL/F (L/h), mean (SD)	17.9 (10.6)	9.2 (6.1)^b^
*V_z_*/*F* (L), mean (SD)	1159 (573.9)	951.3 (581.8)^b^
CL_R_ (L/h), mean (SD)	0.122 (0.105)^c^	0.094 (0.071)^d^
AZ5104		
AUC (nmol/L·h), geometric LS mean (%GCV)	1202 (79.7)	1953 (68.7)^e^
Comparison, ratio, % (90% CI)	162.5 (81.5, 323.8)	
*C*_max_ (nmol/L), geometric LS mean (%GCV)	10.15 (59.0)	8.76 (76.6)
Comparison, ratio, % (90% CI)	86.3 (49.3, 151.2)	
*t*_max_ (h), median (min, max)	35.9 (6.0, 48.4)	24.2 (24.0, 49.3)
*t*_1/2λz_ (h), mean (SD)	59.0 (18.2)	74.3 (17.8)^e^
MRC_max_, mean (SD)	0.057 (0.027)	0.040 (0.013)
MRAUC, mean (SD)	0.119 (0.059)	0.096 (0.020)^e^
CL_R_ (L/h), mean (SD)	0.516 (0.425)^c^	0.141 (0.018)^d^
AZ7550		
AUC (nmol/L·h), geometric LS mean (%GCV)	563.0 (28.1)^b^	417.7 (17.8)^f^
Comparison, ratio, % (90% CI)	74.2 (53.0, 103.9)	
*C*_max_ (nmol/L), geometric LS mean (%GCV)	4.56 (47.9)	2.62 (58.9)
Comparison, ratio, % (90% CI)	57.5 (36.4, 90.8)	
*t*_max_ (h), median (min, max)	24.0 (9.92, 71.8)	24.3 (6.00, 72.8)
*t*_1/2λz_ (h), mean (SD)	71.1 (18.2)^b^	83.2 (9.63)^f^
MRC_max_, mean (SD)	0.0255 (0.0125)	0.0124 (0.0056)
MRAUC, mean (SD)	0.0753 (0.0266)^b^	0.0273 (0.0214)^f^
CL_R_ (L/h), mean (SD)	0.858 (0.649)	0.327 (0.0946)^d^

^a^ Normal renal function creatinine clearance (CrCL) ≥90 mL/min; severe renal impairment CrCL <30 mL/min. One patient who had a CrCL of 80 mL/min at screening was excluded from this summary.

^b^ n = 6.

^c^ n = 7.

^d^ n = 4.

^e^ n = 5.

^f^ n = 3.

Terminal half‐life (*t*_1/2λZ_) and time of maximum concentration (*t*_max_) were longer in patients with SRI relative to patients with NRF Table 2. Apparent volume of distribution (*V_z_*/*F*) and apparent plasma clearance (CL/F) were lower in patients with SRI than in patients with NRF (arithmetic mean). Renal clearance (CL_R_) was low in both groups, and represented approximately 1% or less of CL/F.

CrCL accounted for less than 18% of the between‐patient variability in osimertinib exposure and the slopes of the log‐linear regression of osimertinib exposure vs CrCL were not statistically different from zero (*C*_max_ *P* = .5574, AUC *P* = .1151) showing no relationship between osimertinib exposure and CrCL.

### 3.3 Osimertinib metabolite pharmacokinetic parameters

The metabolite to parent ratios for *C*_max_ and AUC were similar for patients with SRI and for patients with NRF, and overall amounted to less than 11% of osimertinib exposure Table 2. CrCL accounted for <17% and <35% of the between‐patient variability in AZ5104 and AZ7550 exposure, respectively. We found no relationship between AZ5104 or AZ7550 exposure and CrCL. AZ5104 exposure based on *C*_max_ and AUC was approximately 0.86‐fold and 1.62‐fold, respectively, and for AZ7550, it was approximately 0.57‐fold and 0.74‐fold, respectively, for patients with SRI relative to patients with NRF Table 2.

### 3.4 Population pharmacokinetic analysis

**FIGURE 3:**
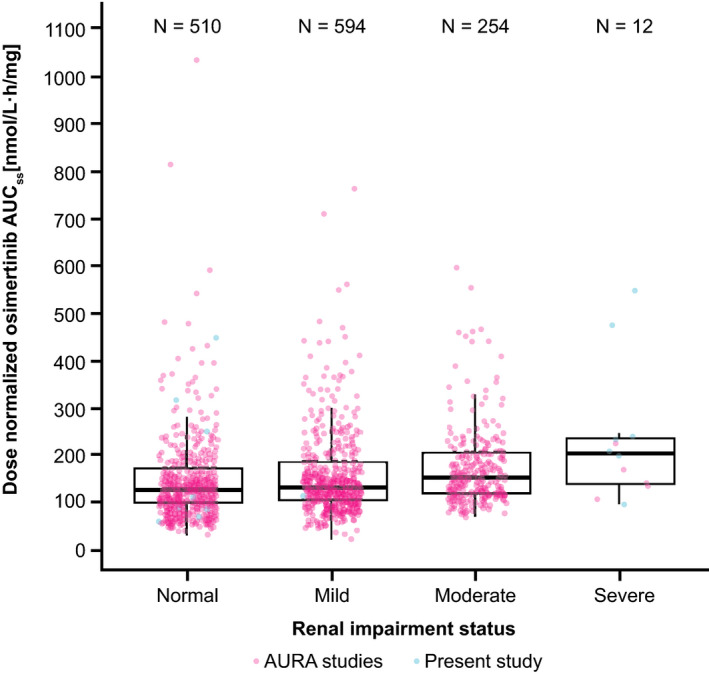
Osimertinib AUC as a function of renal impairment. Circles represent individual AUC_ss_ values based on population PK analysis in AURA studies; for the present study, individual AUC values are shown. AUC, area under the concentration‐time curve; AUC_ss_, area under the plasma concentration‐time curve at steady state; PK, pharmacokinetics

**FIGURE 4:**
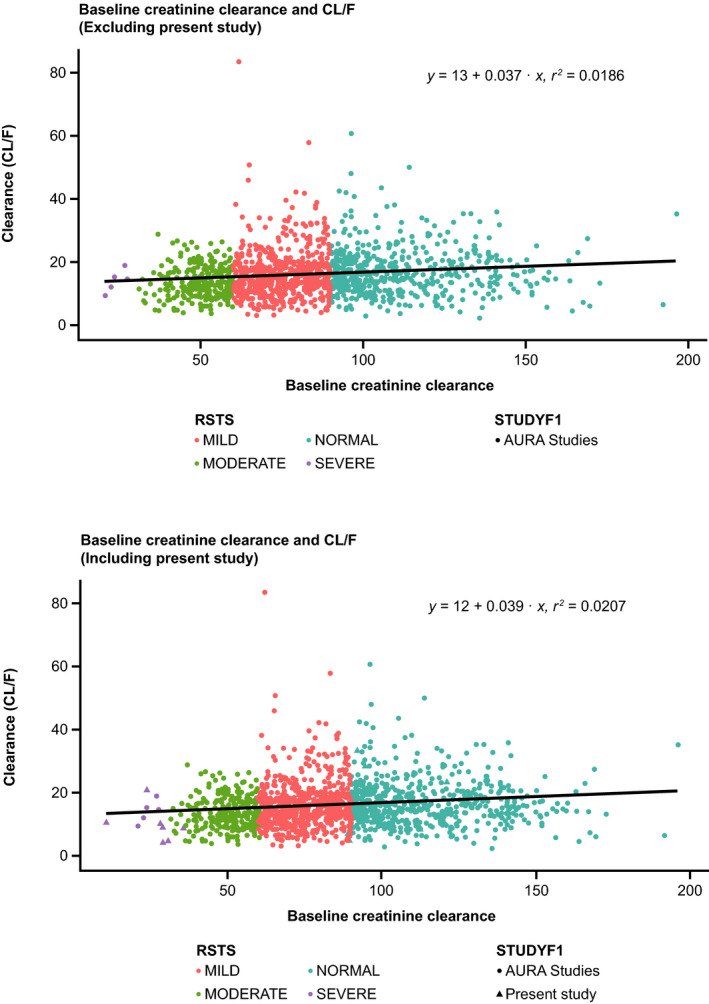
Individual osimertinib AUC function of baseline creatinine clearance (CrCL) in patients across the clinical program. Circles represent individual AUC values; based on population PK analysis. AUC_ss_, area under the plasma concentration‐time curve at steady state; CrCL, creatinine clearance; PK, pharmacokinetics; RSTS, renal impairment status

A graphical and a statistical analysis was performed which included 12 patients with SRI (seven from the present clinical study and five from across clinical studies, of a total of ~1400 patients) and compared to the patients with NRF. The population PK model‐derived osimertinib AUC was plotted as a function of renal impairment. Inclusion of the present results along with the population PK analysis results showed that the data in the present study had a similar range of exposures Figure 3. Individual osimertinib AUC_ss_ was plotted as a function of baseline CrCL which showed no clear relationship between baseline CrCL and osimertinib exposure Figure 4. A comparison of osimertinib PK parameters after single‐dose administration across the clinical study program is shown in Supplementary Section and Table S2.

The linear regression analysis showed that the correlation between CL/F (total apparent clearance) and baseline CrCL is weak (*R* ^2^ less than .02) and this correlation appears to be similar while including and excluding data from this study. This suggests that the results of this study are consistent with the results from population PK analysis regarding the influence of CrCL to the overall clearance of osimertinib.

A combined statistical analysis of 12 patients with SRI vs NRF (n = 508) shows that osimertinib exposure, based on AUC_ss_, was 1.48‐fold higher for patients with SRI. This was lower than the 1.85‐fold increase in patients with SRI, which was observed based on this study alone.

A matched comparison analysis confirmed that the increase in exposure observed with SRI shows a median of 1.26‐fold increase in patients with SRI, vs patients with NRF, when a random dataset of 12 patients with NRF were selected from the population PK dataset (age, sex, and body weight were matched as per study criteria) Table 3.

**TABLE 3:** Population PK analysis: Statistical comparison of AUC_ss_ pharmacokinetic parameter

Parameter (unit)	Renal function group	N^a^	Geometric mean	Comparison of patients with severe renal impairment vs normal patients		Matched comparison of patients with severe renal impairment vs normal patients		
				Pair	Ratio (90% CI)	Ratio^b^ (95% CI)	Lower bound^c^ (95% CI)	Upper bound^d^ (95% CI)
AUC_ss_ (nmol/L·h/mg)	Normal	508	132	Severe vs Normal	1.5 (1.1, 2.0)	1.3 (1.1, 1.5)	0.9 (0.7, 1.0)	1.9 (1.6, 2.3)
	Severe	12	201					

Note

Relationship between renal function (CrCL calculated on baseline) and natural log‐transformed osimertinib PK parameters AUC_ss_ is presented here.

Results are based on an ANOVA model with a fixed effect for renal function group.

*Abbreviations*: ANOVA, analysis of variance.

^a^ Three subjects without PK exposure are not included in this analysis.

^b^ Ratio: Median ratio of 100 matched datasets.

^c^ Lower bound: Median of 5% CI obtained for each 100 matched datasets.

^d^ Upper bound: Median of 95% CI obtained for each 100 matched datasets.

### 3.5 Safety

In Part B, the mean total treatment duration was 53.7 days (standard deviation [SD], 35.18), with a median of 64.5 days (range, 5‐84). The mean actual treatment duration was 51.5 days (SD, 33.45), with a median of 62.0 days (range, 5‐84).

In Part A, 33% (3 of 9) of patients in the normal group and 57% (4 of 7) of patients in the severe impairment group experienced at least one AE. The most commonly reported were: nausea, vomiting, and weight decrease (reported for one patient in each group). In Part B, all six (100%) patients experienced an AE with anemia and asthenia most commonly reported (three patients each). Most of the AEs reported during the study were mild or moderate in severity. In Part A, grade ≥3 AEs were reported in two (13%) patients (one patient in each group; hypotension [one patient in normal group], constipation and hypertension [one patient in severe group]), none of which were considered by the investigator to be related to treatment with osimertinib. In Part B, grade ≥3 AEs were reported in four (67%) patients: asthenia (two patients), anemia (two patients), and renal failure (one patient). All grade ≥3 AEs were considered by the investigator to be unrelated to treatment with osimertinib (Supplementary Section and Table S3). AEs of special interest reported in Parts A and B are summarized in Supplementary Section and Table S3. With the exception of diarrhea (reported for two patients in each part), patients with AEs of special interest were reported singularly. Serious adverse events (SAEs) were reported in two patients (both with SRI, 29%) in Part A (rib fracture and hypotension) and in two patients (33%) in Part B (dyspnea and asthenia). The investigator did not consider any of the SAEs to be possibly related to osimertinib.

No AEs of interstitial lung disease and no AEs leading to death were reported in either part of the study. Three patients (two patients in Part A and one patient in Part B) died due to disease progression. There were no clinically significant trends observed in vital signs, left ventricular ejection fraction assessments, physical examination findings, ECG parameters, or laboratory parameters during the study.

## 4 DISCUSSION

The present study was designed to assess the impact of SRI on the PK of osimertinib in patients with advanced solid tumors. The study was designed in accordance with Food and Drug Administration (FDA) and EMA guidance on the assessment of PK in patients with impaired renal function.^16^, ^20^ In line with the FDA and EMA guidance, a reduced PK study design involving single‐dose PK analysis in patients with SRI and those with NRF was considered appropriate. This was based on the results of prior PK analyses showing that osimertinib and its active metabolites exhibit linear, dose‐proportional (20‐240 mg) and time‐independent PK with steady state predictable from a single dose.^10^, ^17^ Furthermore, based on the population PK analysis of patients across the osimertinib clinical studies, mild‐to‐moderate renal impairment had no impact on the plasma clearance of osimertinib, therefore it was considered unlikely that SRI would have a significant impact on the PK of osimertinib.^17^ Due to limited safety data available in patients with SRI, Part B (3 months’ daily dosing) of the study was included to enable further understanding of the safety characteristics of this patient population. A comprehensive review of the concomitant medications showed no patients were taking strong inducer of CYP3A4/5 or any other drug that could impact the analysis during the PK phase of the study.

Our results showed that osimertinib and AZ5104 exposure in patients with SRI, based on AUC, was 1.85‐fold and 1.62‐fold higher relative to that of patients with NRF. In contrast, AZ7550 AUC was approximately 0.74‐fold relative to patients with NRF. Osimertinib *C*_max_ in patients with SRI was 1.19‐fold higher while AZ5104 and AZ7550 *C*_max_ were 0.86‐fold and 0.57‐fold relative to patients with normal function. For all analytes, between‐patient variability in exposure was high; the 90% CIs in the ANOVA comparison were wide and, with the exception of AZ7550 *C*_max_, all 90% CIs included unity. Renal clearance for all three analytes was negligible in patients with SRI and in patients with NRF. Hence, changes in renal clearance are unlikely to account for the higher exposure observed in SRI patients. Similar to other studies which have shown renal impairment to affect the function of CYP3A enzymes, here the changes to AZ5104 and AZ7550 (which are primarily metabolized by CYP3A) appear to be qualitatively similar to that seen with the strong CYP3A inhibitor, itraconazole.^21^, ^22^

The mean exposure to osimertinib and its metabolites (AZ5104 and AZ7550) in patients with NRF in this study was comparable to that observed in patients in other studies with advanced NSCLC (mean AUC of 11 070 nmol/L·h in this study and 10 590‐13 520 nmol/L·h in other studies; Supplementary Section Table S2) and in the population PK analysis (mean AUC_ss_ 11 258 nmol/L·h).^17^ While exposure to osimertinib was higher in the cohort with SRI, a correlation between osimertinib and/or metabolite(s) exposure with CrCL could not be established. Several individual patients with high osimertinib AUC values were also observed in the NRF population in this study, similar to those observed in prior studies (maximum AUC values: 74 500 nmol/L⋅h [NCT02161770], 25 500 nmol/L⋅h [NCT02163733], and 40 400 nmol/L⋅h [NCT02157883]). Furthermore, the upper range of osimertinib *C*_max_ observed in this study was similar in the two populations and below that observed under single‐dose conditions in several other trials (1260 nmol/L [NCT02161770], 704 nmol/L [NCT02157883], and 803 nmol/L [NCT02908750]). The high individual exposure to osimertinib in this study observed in two patients with AUC above 35 000 nmol/L⋅h could not be explained by their demographic and/or disease characteristics and suggests variability of exposure of osimertinib. In patients with SRI, the higher osimertinib exposure cannot be explained by changes in protein binding. In general, renal impairment may be associated with an increase in unbound drug concentrations, which would lead to increase in drug clearance as it is the unbound drug that is cleared from the body, the expected net effect, if any, would be a decrease not an increase in total drug exposure. Neither osimertinib nor its metabolites (AZ5104 and AZ7550) were quantifiable in urine and plasma ultrafiltrate in any of the samples evaluated. Considering the detection limit and the concentrations observed in plasma for each analyte, free fractions as low as <0.01% to 0.1%, 0.2% to 1.7%, and 0.7% to 9.3% could have potentially been detected for osimertinib, AZ5104 and AZ7550, respectively. This suggests that all three analytes were present almost entirely in the bound form in patients with SRI and in patients with NRF, which indicates further that SRI did not alter the level of binding for osimertinib and its metabolites (AZ5104 and AZ7550) to a degree to detect unbound concentrations in urine and plasma ultrafiltrate.

Osimertinib and metabolites are minor substrates for p‐glycoprotein and breast cancer resistance protein; however, p‐glycoprotein and/or breast cancer resistance protein transport is unlikely to be of clinical relevance given the osimertinib concentrations following an 80 mg dose, as these transporters are expected to be saturated in the intestinal compartment at that dose. The higher exposure to osimertinib observed in SRI patients is unlikely to be a result of renal disease mediated changes in these transporters.

In patients with SRI, the higher osimertinib exposure may possibly be due to the limited number of patients evaluated in this study (seven in this study and five in the population PK analysis, of a total of ~1400). This was due to difficulties in recruiting eligible patients and is a common limitation of such trials. Guidance from the EMA suggest that a population of 6‐8 patients per group is required to provide adequate PK data,^16^ thus, while the study population was small, it was sufficient to assess the effects of SRI on the PK of osimertinib and the results we observed were reflected in the overall population PK population.

Due to the limited number of patients with SRI, linear regression and statistical analysis with the inclusion of all patients, and a matched comparison analysis, were performed. All of these analyses clearly show that the effect of renal impairment is higher in this study vs all other analyses, which might be a reflection of limited number and higher variability. It should be noted that osimertinib shows a dose proportional increase, with the exposure at 160 mg being 2‐fold higher than the exposure at 80 mg. Therefore, the 1.48‐1.85‐fold increase in exposure in patients with SRI using all patients with SRI is lower than the exposure observed at the 160 mg dose. A matched comparison analysis indicates that the likely effect of SRI is 1.26‐fold when 100 clinical trials were simulated.

Despite the small number of patients in each cohort, there were no apparent differences in safety observed between patients with NRF and patients with SRI. The number and type of AEs reported in our study were consistent with expectations for this patient population and the current known safety profile for osimertinib. Moreover, no safety signals were identified during or after 12 weeks of continuous exposure to osimertinib in patients with SRI.

Renal impairment did not meaningfully alter AZ5104 metabolite/parent ratios for *C*_max_ and AUC, which accounted for less than 11% of osimertinib exposure. AZ7550 metabolite/parent ratios for *C*_max_ and AUC were lower in patients with SRI; metabolite AUC amounted to less than 2% (SRI) or 8% (NRF) of the exposure to osimertinib. As AZ7550 accounts for <10% of the exposure of osimertinib with similar in vitro pharmacological properties, the changes in AZ7550 metabolite/parent exposure ratio are not considered of clinical relevance.

Based on the AURA phase I study, clinical activity was demonstrated at all doses studied (20 to 240 mg in T790M population and at both 80 and 160 mg in the first‐line population), with no maximum tolerated dose reached at the 240 mg dose.^11^, ^17^ It is also clinical practice to reduce osimertinib dose to 40 mg to manage drug‐related toxicities, while keeping adequate osimertinib efficacy dose levels. As such, it has been established that increases in mean osimertinib exposure of less than 2‐fold (ie, less than exposure equivalent to a 160 mg dose) and decreases of no more than 50% (ie, greater than that achieved at a 40 mg dose) would require no dose adjustments as it will unlikely have any clinically meaningful impact on efficacy or safety.^17^ Osimertinib shows inter‐patient PK variability on CL/F (45% between subject variability)^23^ and, as such, any changes in exposure that is less than 2‐fold (lower than that observed with the 160 mg dose) is unlikely to alter the benefit:risk ratio.

In conclusion, mean osimertinib PK exposure was higher in patients with SRI vs patients with NRF. However, a lack of correlation between exposure and the CrCL, together with a similar and consistent known safety profile of osimertinib after single and multiple dosing in patients with SRI, indicate that no dose adjustment for osimertinib is required when treating patients with SRI. Nevertheless, as the mean exposure change due to SRI approached an almost 2‐fold increase in the present study, a proper clinical assessment and continuous monitoring in patients with severe and end stage renal disease should be considered.

## DATA SHARING AND ACCESSIBILITY

Data underlying the findings described in this article may be obtained in accordance with AstraZeneca's data sharing policy described at https://astrazenecagrouptrials.pharmacm.com/ST/Submission/Disclosure.

## DISCLOSURES

Karthick Vishwanathan, Marcello Marotti, and Martin Johnson are AstraZeneca employees and shareholders. Doris Weilert is an employee of IQVIA, formerly Quintiles, the contract research organization that conducted the trial. Andrew Mills is a contract employee of AstraZeneca. Inmaculada Sanchez‐Simon, Maria de Miguel‐Luken, Bhumsuk Keam, Nicolas Penel, and Alain Ravaud have nothing to disclose.

## AUTHOR CONTRIBUTIONS

KV, DW, MM, and AM participated in research design. IS‐S, BK, NP, MdML, MJ, and AR conducted experiments. KV, IS‐S, BK, NP, MdML, DW, AM, MM, MJ, and AR contributed new reagents or analytic tools. KV, DW, AM, MM, MJ, and AR performed data analysis. KV, IS‐S, BK, NP, MdML, DW, AM, MM, MJ, and AR wrote or contributed to the writing of the manuscript.

## Supporting information

Supplementary Material
