# Supplementary material for: A multicenter, phase I, pharmacokinetic study of osimertinib in cancer patients with normal renal function or severe renal impairment
Source: Pharmacol Res Perspect. 2020 Jun 22;8(4):e00613. doi: 10.1002/prp2.613 (PMC7307240; doi:10.1002/prp2.613)
Supplement: Supplementary file 1 — Supplementary Material [file PRP2-8-e00613-s001.docx]

Pharmacology Research and Perspectives

A multicentre, phase I, pharmacokinetic study of osimertinib in cancer patients with normal renal function or severe renal impairment

Karthick Vishwanathan, Inmaculada Sanchez-Simon, Bhumsuk Keam, Nicolas Penel, Maria de Miguel-Luken, Doris Weilert, Andrew Mills, Marcello Marotti, Martin Johnson, Alain Ravaud

# Supplementary Section

**Estimated creatinine clearance (CrCL) by the Cockcroft-Gault (C-G) equation**

CrCL in mL/min was estimated from a spot serum creatinine (mg/dL) determination using the following formula:

$$CrCL \left( \frac{mL}{min} \right)=\left( \frac{\left[ 140-age \left( years \right) \right]\times weight \left( kg \right)}{72 \times serum creatinine \left( \frac{mg}{dL} \right)} \right)\{\times0.85 for female patients\}$$

**Supplementary Methods**

**Participants**

Treatment with another EGFR-TKI had to be completed 8 days or ~5x half-life, whichever was the longer, before entering; any cytotoxic chemotherapy or other anticancer drugs had to be completed within 14 days of study treatment; major surgery had to be completed within 4 weeks of study treatment; radiotherapy had to be completed within 1 week of study treatment. Throughout the study, all patients had to avoid concomitant use of medications, herbal supplements and/or ingestion of foods with known potent inducer effects on CYP3A4. For statins, patients taking rosuvastatin had creatine phosphokinase levels monitored (due to BCRP-mediated increase in exposure).

**Sample collection and bioanalysis**

In brief, calibration, quality control and clinical study samples (40 μL) were spiked with an internal standard (13C,2H3-osimertinib), processed by either protein precipitation (plasma method) or dilution (urine and plasma ultrafiltrate) and then simultaneously assayed for osimertinib, AZ5104 and AZ7550 using reversed-phase high performance liquid chromatography with Turbo Ion Spray® tandem mass spectrometric detection (AB Sciex, Framingham, MA, USA). Drug-to-internal standard peak area ratios for the standards were used to create either a linear or quadratic calibration curve using 1/x2 weighted least-squares regression analysis. Concentrations of each analyte were quantified by comparing the ratios for each in trial samples with those in the relevant calibration curve. No analytically significant interferences from endogenous matrix components were observed at the retention times of each analyte in the matrix samples screened. All methods demonstrated acceptable selectivity with mean, normalized matrix factors of 1.00 ± 0.08 being observed at the concentrations tested. The lower limit of quantification was 1.00 nM for osimertinib and 1.03 nM for AZ5104 and AZ7550 in all matrices. The accuracy ranged from 94% to 111%, and precision ranged from 2% to 12% for all analytes across all matrices.

**Statistical methods**

PK parameters for plasma osimertinib and its metabolites were summarized using appropriate descriptive statistics. Natural log-transformed AUC, AUC from zero to last quantifiable concentration at time (AUC0–t), and Cmax were compared between renal function groups (severe vs normal) separately for osimertinib and each metabolite using an analysis of variance model, with renal function group as a fixed effect. Estimates of the mean difference between renal function groups (severe/normal) and corresponding 90% confidence intervals (CIs) were calculated. The mean differences and the CIs were back-transformed to the original scale to give estimates of the ratios (severe vs normal) and the associated 90% CIs. Additionally, back-transformed geometric means together with 95% CIs for AUC and Cmax were estimated and presented for each renal function group. For osimertinib and its metabolites, the relationships between CrCL determined at baseline (on Day -1 or predose Day 1) and natural log-transformed PK parameters AUC and Cmax, were explored using a regression model with CrCL value included as the independent variable and PK parameter as the dependent variable. Slopes, intercepts and the associated 90% CIs were presented along with the coefficient of determination.

**Supplementary Results**

**Protocol deviations**

A total of three patients (all in the normal group) had important protocol deviations during the study. Two patients had deviations relating to laboratory assessment criteria. Laboratory samples were not taken on Day 10 for one patient due to a worsening of status. For the second patient, the site obtained two samples of blood for PK analysis (instead of the required one sample) as the kit for Day 3 was used. Data for both patients were included in the PK analysis. A third patient had a deviation relating to eligibility and entry criteria. The patient was enrolled into the study even though inclusion criterion #2 of CrCL ≥90 mL/min calculated using the protocol-specified C-G formula was not met (the site calculated CrCL with a corrected C-G formula instead of the C-G formula specified in the protocol). This patient’s data were listed and summarized with the normal renal function cohort in the demographic and safety summaries and the patient’s osimertinib and metabolite concentrations and calculated PK parameters were listed. However, PK results for this patient were excluded from all categorical (by renal function) analyses for primary and secondary PK endpoints. The patient’s data were included in the exploratory regression analysis of exposure data (AUC and C_max_) vs CrCL, since CrCL was treated as a continuous variable in this analysis.

**Results**

**Table S1:** Classification of renal function based on estimated creatinine clearance.

| **Stage** | **Description^a^** | **CrCL^b^ (mL/min)** |
| --- | --- | --- |
| 1 | Control (normal) GFR | ≥90 |
| 2 | Mild decrease in GFR | 60–89 |
| 3 | Moderate decrease in GFR | 30–59 |
| 4 | Severe decrease in GFR | 15–29 |
| 5 | End Stage Renal Disease | <15 not on dialysis |
|  |  | Requiring dialysis |

^a^Stages of renal impairment are based on *K/DOQI Clinical Practice Guidelines for Chronic Kidney Disease (CKD) from the National Kidney Foundation* in 2002.[24]

^b^CrCL: estimated creatinine clearance based on the Cockcroft-Gault formula.

CrCL, creatinine clearance; GFR, glomerular filtration rate.

**Table S2:** Description of studies included in the population PK dataset.

| **Study number** | **Study description** | **Number of patients with PK information** | **PK sampling schedule^a^** |
| --- | --- | --- | --- |
| NCT01802632 (AURA) | Phase 1 component in EGFR mutation positive advanced NSCLC patients.  Doses: 20, 40, 80, 160 and 240 mg (capsule) 80 mg (tablet)  Note: capsule and tablets provided similar exposure and formulation has no impact on osimertinib exposure | 402^b^ | Following a single dose: pre-dose, (0.5), 1, 1.5, 2, (3), 4, 6, 8, 10, 12, 24, 48, (72) and (120) hours post dosing  Following multiple doses: Cycle 1 Day 8: pre-dose, 0.5, 1, 1.5, 2, 3, 4, 6, 8, 10, 12 and 24 hours post dosing, or Cycle 2 Day 1: pre-dose, 1, 1.5, 2, 4, 6, 8, 10, 12 and 24 hours post dosing and pre-dose on Cycle 1 day 15 |
| NCT01802632 (AURA extension) | Phase 2 in EGFR T790M positive advanced NSCLC patients who have progressed following either one prior therapy with an EGFR-TKI agent or following treatment with at least one EGFR-TKI and at least one prior platinum-based doublet chemotherapy. Dose: 80 mg (tablet) | 201 | Cycle 2 Day 1: pre-dose, 1, 1.5, 2, 4, 6, 8, 10, 12 and 24 hours post dosing and pre-dose on Cycle 1 days 1, 8 and 15 |
| NCT02094261 (AURA2) | Phase 2 component in EGFR T790M positive advanced NSCLC patients who have progressed following either one prior therapy with an EGFR TKI agent or following treatment with both EGFR TKI and at least one other prior line of therapy, such as cytotoxic doublet chemotherapy or immunotherapy.  Dose: 80 mg (tablet) | 210 | Following a single dose: pre-dose, 1, 2, 4, 6 and 8 hours post dosing  Following multiple doses on Cycle 2 Day 1: pre-dose and on Cycle 3 Day 1: pre-dose, 1, 2, 4, 6, 8, 10, 12 and 24 hours post dosing. Dose reductions were allowed |
| NCT02151981 (AURA3) | A phase 3, open label, randomized study of osimertinib vs platinum-based doublet chemotherapy for patients with locally advanced or metastatic NSCLC whose disease has progressed with previous EGFR-TKI therapy and whose tumors harbor a T790M mutation (AURA3).  Dose: 80 mg (tablet) | 279** | Plasma samples were collected at pre-dose, between 0.5 and 1.5 hours and between 2 and 4 hours after dosing on first day of dosing in cycle 1, 3, 5, 7, 9, 11, and 13. Dose reductions were allowed |
| NCT02296125 (FLAURA) | A phase 3, double-blind, randomized study to assess the efficacy and safety of osimertinib vs a standard of care EGFR-TKI as first-line treatment in patients with EGFRm, locally advanced or metastatic NSCLC.  Dose: 80mg (tablet) | 279 | Plasma samples were collected at pre-dose, between 0.5 and 2 hours and between 3 and 5 hours after dosing at Day 1 cycle 1 and every other cycle thereafter up to and including Cycle 13. Dose reductions were allowed |

| ^a^In AURA the PK sampling scheme was updated based on emerging PK data and hence single dose samples in brackets were not collected in all patients and multiple dose samples were collected on either Cycle 1 Day 8 or Cycle 2 Day 1; multiple dose PK dosing was repeated once daily oral dosing.  ^b^Two patients from each of these studies were not included in the master dataset.  EGFR, epidermal growth factor receptor; EGFRm, EGFR-TKI sensitizing mutation; NSCLC, non-small cell lung cancer; TKI, tyrosine kinase inhibitor. |
| --- |

**Table S3. Summary of adverse events (safety analysis set)**

|  | **Part A** | | **Part B** | **Parts A and B** | |
| --- | --- | --- | --- | --- | --- |
| **Adverse event, n (%)^a^** | Normal renal  function^a^  (N = 9) | Severe renal  impairment^b^  (N = 7) | Severe renal  impairment  (N = 6) | Severe renal  impairment  (N = 7) | Total  (N = 16) |
| Any AE | 3 (33) | 4 (57) | 6 (100) | 6 (86) | 9 (56) |
| Any AE causally related to osimertinib^b^ | 2 (22) | 2 (29) | 3 (50) | 4 (57) | 6 (38) |
| Any AE of CTCAE grade 3 or higher | 1 (11) | 1 (14) | 4 (67) | 5 (71) | 6 (38) |
| Any AE of CTCAE grade 3 or higher causally related to osimertinib^b^ | 0 | 0 | 0 | 0 | 0 |
| Any AE leading to death | 0 | 0 | 0 | 0 | 0 |
| Any SAE (including death) | 0 | 2 (29) | 2 (33) | 4 (57) | 4 (25) |
| Any SAE causing discontinuation of osimertinib | 0 | 0 | 0 | 0 | 0 |
| Any AE leading to discontinuation of osimertinib | 0 | 0 | 1 (17) | 1 (14) | 1 (6) |
| **Adverse events of special interest, n (%)** |  |  |  |  |  |
| Cardiomyopathy | 0 | 0 | 1 (17) | 1 (14) | 1 (6) |
| Diarrhea | 0 | 1 (14) | 1 (17) | 2 (29) | 2 (13) |
| Nail effects* | 0 | 0 | 1 (17) | 1 (14) | 1 (6) |
| Conjunctivitis | 0 | 0 | 1 (17) | 1 (14) | 1 (6) |
| Lacrimation increased | 0 | 1 (14) | 0 | 1 (14) | 1 (6) |
| Eye irritation | 0 | 1 (14) | 1 (17) | 1 (14) | 1 (6) |
| Nephrolithiasis | 1 (11) | 0 | 0 | 0 | 1 (6) |
| Renal failure | 0 | 0 | 1 (17) | 1 (14) | 1 (6) |
| Mucosal dryness | 0 | 0 | 1 (17) | 1 (14) | 1 (6) |

*Grouped terms

^a^Patients with multiple events in the same category are counted only once in that category. Patients with events in more than one category are counted once in each of those categories.

^b^As assessed by the Investigator.

AE counted for Part A if onset was after first dose of osimertinib in Part A and up to and including 30 days post-last dose in Part A (and prior to first dose of osimertinib in either Part B or continued access post-Part A).

AE counted for Part B if onset was after first dose of osimertinib in Part B and up to and including 30 days post-last dose in Part B (and prior to first dose of osimertinib in continued access post-Part B).

Subjects in Part B received osimertinib 80 mg once daily for 12 weeks.

AE, adverse event; CTCAE, Common Terminology Criteria for Adverse Events; SAE, serious adverse event.

**Figure S1. Individual and geometric mean of osimertinib for each renal**

**function group^a^ (pharmacokinetic analysis set): A. AUC; B. C_max_.**

**A.**
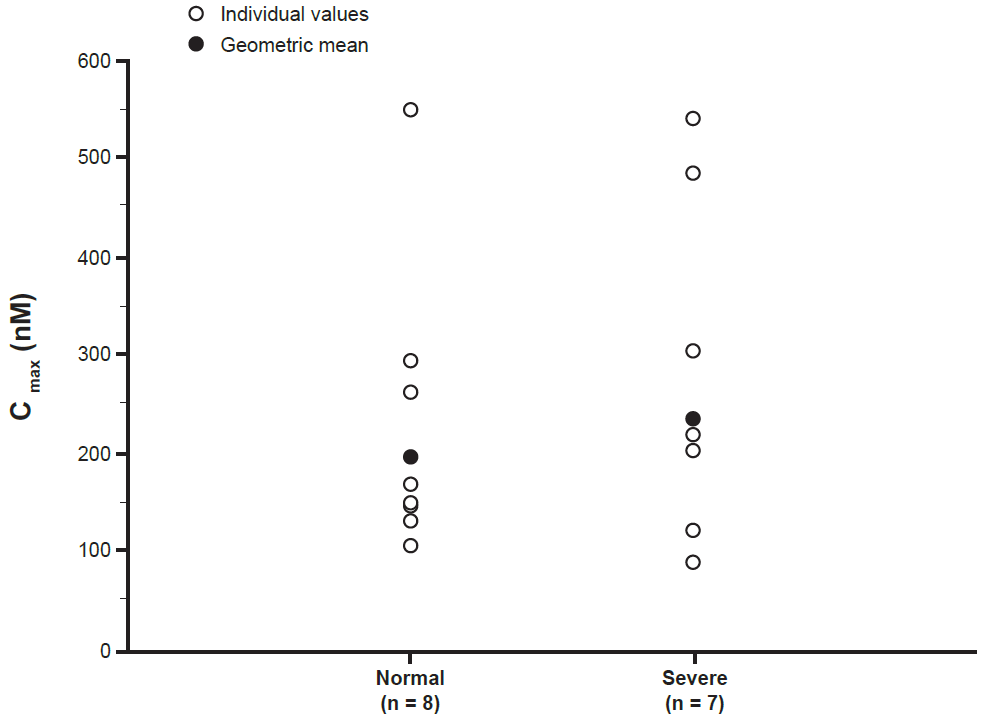


**B.**
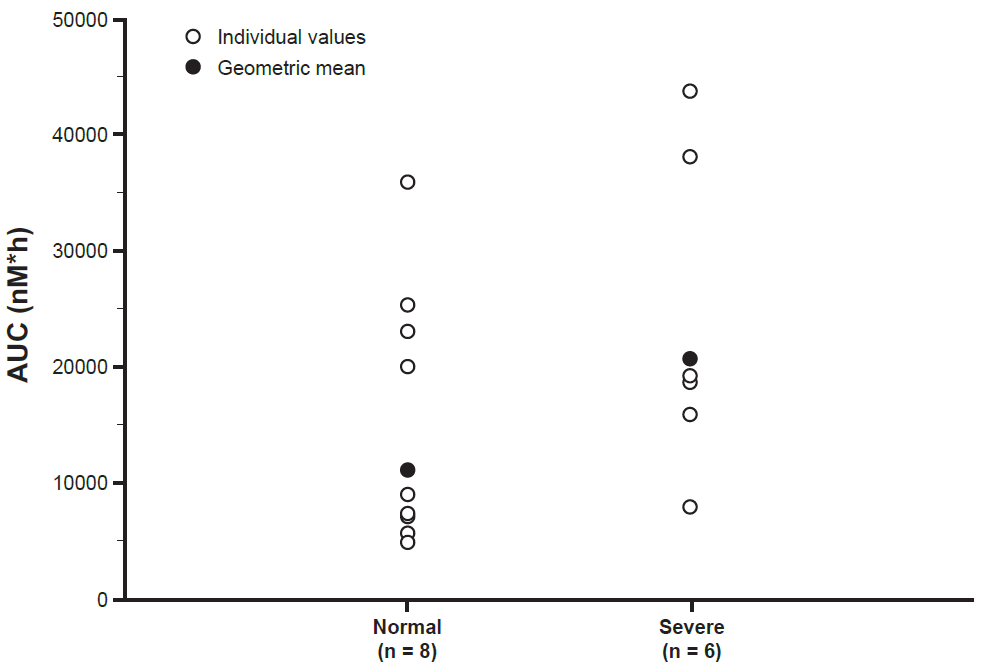


^a^Normal renal function creatinine clearance (CrCL) ≥90 mL/min; severe renal impairment CrCL of <30 mL/min. One patient who had a CrCL of 80 mL/min at screening was excluded from this summary. AUC, area under plasma concentration-time curve; C_max_ maximum plasma drug concentration.
